# Behavioural digital biomarkers enable real-time monitoring of patient-reported outcomes: a substudy of the multicentre, prospective observational SafeHeart study

**DOI:** 10.1093/ehjqcco/qcad069

**Published:** 2023-12-06

**Authors:** Maarten Z H Kolk, Diana M Frodi, Joss Langford, Caroline J Meskers, Tariq O Andersen, Peter Karl Jacobsen, Niels Risum, Hanno L Tan, Jesper H Svendsen, Reinoud E Knops, Søren Z Diederichsen, Fleur V Y Tjong

**Affiliations:** Department of Cardiology, Amsterdam UMC, University of Amsterdam, Amsterdam, The Netherlands; Department of Cardiology, Copenhagen University Hospital—Rigshospitalet, Copenhagen, Denmark; Activinsights Ltd, Kimbolton, UK; College of Life and Environmental Sciences, University of Exeter, Exeter, UK; Department of Cardiology, Amsterdam UMC, University of Amsterdam, Amsterdam, The Netherlands; Department of Computer Science, University of Copenhagen, Copenhagen, Denmark; Department of Cardiology, Copenhagen University Hospital—Rigshospitalet, Copenhagen, Denmark; Department of Cardiology, Copenhagen University Hospital—Rigshospitalet, Copenhagen, Denmark; Department of Cardiology, Amsterdam UMC, University of Amsterdam, Amsterdam, The Netherlands; Netherlands Heart Institute, Utrecht, The Netherlands; Department of Cardiology, Copenhagen University Hospital—Rigshospitalet, Copenhagen, Denmark; Department of Clinical Medicine, Faculty of Health and Medical Sciences, University of Copenhagen, Copenhagen, Denmark; Department of Cardiology, Amsterdam UMC, University of Amsterdam, Amsterdam, The Netherlands; Department of Cardiology, Copenhagen University Hospital—Rigshospitalet, Copenhagen, Denmark; Department of Cardiology, Amsterdam UMC, University of Amsterdam, Amsterdam, The Netherlands

**Keywords:** Patient-reported outcome measure, Quality of life, Digital health, Wearable technology

## Abstract

**Aims:**

Patient-reported outcome measures (PROMs) serve multiple purposes, including shared decision-making and patient communication, treatment monitoring, and health technology assessment. Patient monitoring using PROMs is constrained by recall and non-response bias, respondent burden, and missing data. We evaluated the potential of behavioural digital biomarkers obtained from a wearable accelerometer to achieve personalized predictions of PROMs.

**Methods and results:**

Data from the multicentre, prospective SafeHeart study conducted at Amsterdam University Medical Center in the Netherlands and Copenhagen University Hospital, Rigshospitalet in Copenhagen, Denmark, were used. The study enrolled patients with an implantable cardioverter defibrillator between May 2021 and September 2022 who then wore wearable devices with raw acceleration output to capture digital biomarkers reflecting physical behaviour. To collect PROMs, patients received the Kansas City Cardiomyopathy Questionnaire (KCCQ) and EuroQoL 5-Dimensions 5-Level (EQ5D-5L) questionnaire at two instances: baseline and after six months. Multivariable Tobit regression models were used to explore associations between digital biomarkers and PROMs, specifically whether digital biomarkers could enable PROM prediction. The study population consisted of 303 patients (mean age 62.9 ± 10.9 years, 81.2% male). Digital biomarkers showed significant correlations to patient-reported physical and social limitations, severity and frequency of symptoms, and quality of life. Prospective validation of the Tobit models indicated moderate correlations between the observed and predicted scores for KCCQ [concordance correlation coefficient (CCC) = 0.49, mean difference: 1.07 points] and EQ5D-5L (CCC = 0.38, mean difference: 0.02 points).

**Conclusion:**

Wearable digital biomarkers correlate with PROMs, and may be leveraged for real-time prediction. These findings hold promise for monitoring of PROMs through wearable accelerometers.

## Graphical Abstract

**Graphical Abstract:**
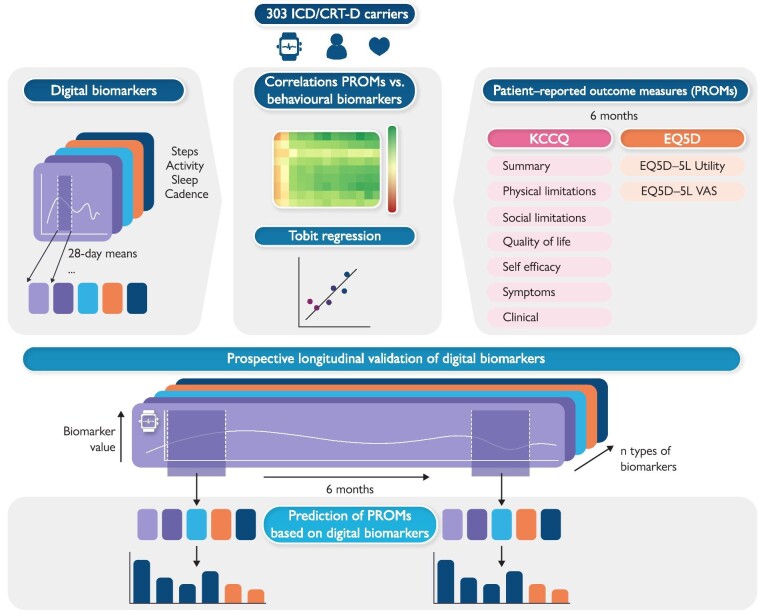

Key learning points **What is already known:**

- Patient-reported outcome measures (PROMs) are essential for patient-centred care and treatment evaluation, reflecting patients’ quality of life and treatment experiences. However, frequent PROM administration comes with limitations, including recall and non-response bias, increased respondent burden, and missing data.
- Digital health technologies, particularly wearable sensors, facilitate the collection of high-density, patient-centric digital biomarkers, and are increasingly being utilized in clinical studies to continuously quantify an individual's physical behaviour.
- Several studies have explored the correlations between physical activity and PROMs. We hypothesized that behavioural digital biomarkers can be utilized to estimate PROMs over time and enable personalized PROM monitoring.

**What this study adds:**

- Significant associations were observed between behavioural digital biomarkers and PROMs, along with moderate correlations between the observed and predicted values derived from the prediction models, particularly for PROMs assessing physical and social limitations, frequency and severity of symptoms, and quality of life.
- Integration of a prediction model that provides personalized PROM predictions and alarms clinicians if a clinically important difference is expected could enable clinicians to proactively approach individuals to collect PROM data and tailor patient care.
- This study provides a proof of concept for personalized PROM monitoring using behavioural digital biomarkers obtained from wearable devices.

## Introduction

Clinical patient care is transformed by digital health technologies, such as wearable sensors, that enable collection of high-density, quantitative, patient-centric digital biomarkers.^1^ In particular, wearable accelerometers have been increasingly used in clinical studies for continuous quantification of physical behaviour using a collection of digital biomarkers that reflect all activities an individual undertakes throughout the day and night.^2^ Digital phenotyping, which describes the passive and unobtrusive collection of objective data typically using accelerometers, has the potential to reveal behavioural aspects related to clinically relevant outcomes.^3^ Behavioural digital biomarkers such as duration and intensity of daily activities, postural changes, sleep behaviour, and rest–activity patterns have been previously associated with functional decline, mental health outcomes, disease progression, and incident disease.^4–6^ Alongside the utilization of objective behavioural and physiological measures for patient monitoring, there is an increasing emphasis on considering the patient's perspective through patient-reported outcome measures (PROMs).^7^ PROMs are acknowledged as fundamental for patient-centred care and treatment evaluation, by reflecting the patient's perception of their quality of life, functional status, and treatment experiences.^8,9^ Furthermore, PROMs form the basis of health technology assessments that consider health-related quality of life to assess the (cost-)effectiveness of treatments and inform regulatory decisions.^10^ Digital technologies have lowered the threshold for administration of PROMs, which comes with limitations such as recall and non-response bias, increased respondent burden, and missing data.^11–13^ A few studies have described correlations between physical activity and PROMs,^14–20^ but it is unclear whether behavioural digital biomarkers could enable estimation of PROMs over time. The aim of this study was to assess the potential of digital biomarkers to predict PROMs, and provide a proof of concept for personalized PROM monitoring.

## Methods

### Study design

This is a substudy of the ongoing SafeHeart study, a multicentre, prospective observational study, conducted at Amsterdam University Medical Center in the Netherlands and Rigshospitalet in Copenhagen, Denmark. The purpose of this study was to develop a personalized model to predict therapy from an implantable cardioverter-defibrillator (ICD) therapy for malignant ventricular arrhythmias.^21^ Data used to create the prediction model included recordings from a wearable accelerometer. All participants were enrolled between May 2021 and September 2022. Patient inclusion was conducted through telephone-based procedures, and enrolment date was defined as the day when the wearable device was delivered to the patient. Ethical approval for the study protocol was obtained from the ethics board at both study sites, and all participants provided informed consent prior to their enrolment. Throughout the study, participants had the option to withdraw their participation at any stage, either partially (by discontinuing the use of the wearable device) or completely. We adhered to the *Strengthening the Reporting of Observational Studies in Epidemiology* reporting guidelines for observational studies.^22^ The study was registered at the National Trial Registration in the Netherlands (Trial NL9218; https://www.trialregister.nl).

### Participants

Patients were eligible for inclusion in the SafeHeart study if they met the following criteria: (i) implantation of an ICD with or without cardiac resynchronization therapy (CRT-D) within five years prior to enrolment, (ii) appropriate or inappropriate ICD therapy or evidence of ventricular arrhythmias within eight years prior to enrolment, (iii) participation in a remote monitoring programme, and (iv) minimum age of 18 years old. Serious physical disability, end-stage heart failure, and a life expectancy <1 year were among the exclusion criteria. The full list of criteria was published elsewhere.^21^

### Patient-reported outcome measures

Two PROMs were used for this analysis: the generic EuroQoL 5-Dimensions 5-Levels (EQ5D-5L) questionnaire and disease-specific Kansas City Cardiomyopathy Questionnaire (KCCQ).^23–25^ The EQ5D-5L measures health states across five domains: mobility, self-care, the ability to carry out usual activities, pain/discomfort, and anxiety/depression rated on a five-point Likert scale.^24^ The EQ5D-5L utility score for health-related quality of life ranges between −0.590 (the worst score) and 1.000 (the best score). In addition, participants rate their health from 0 (worst possible) to 100 (best health imaginable) on a visual analogue scale (EQ-VAS). Using a set of weights (value sets), the EQ5D-5L health states were converted into a summary index value. Second, the KCCQ is a 23-item questionnaire that has been specifically designed to monitor the quality of life and health status of heart failure patients. The 23 questions of the KCCQ map to five domains: symptoms, physical limitation, social limitation, quality of life, and self-efficacy. From the KCCQ, scores on a scale from 0 (worst health state) to 100 (perfect health) can be calculated, which can be averaged to compute the clinical score and the summary score. Scores from 0 to 24 represent very poor to poor health status, 25 to 49 poor to fair, 50 to 74 fair to good, and 75 to 100 good to excellent. The minimum clinically important difference (MCID) of the KCCQ, defined as a meaningful change in the questionnaire score that has clinical significance for patients, is five points.^25^

### Digital biomarkers: wearable accelerometry

Accelerometer-enabled devices allow for continuous and objective quantification of daily physical behaviour by the recording of body movement along reference axes and signal analysis (e.g. intensity, frequency, and volume of activity and postural changes). In this study, various behavioural metrics were collected, including activity and inactivity counts, duration of activity and inactivity episodes, activity intensity, activity volume, step count (total, slow, and fast), cadence, sleep duration, sleep efficiency, wake up after sleep onset (WASO), nap duration, and sleep onset latency. A complete overview of the collected metrics and their definitions is displayed in *Table 1*. To collect these metrics, participants wore the GENEActiv accelerometer (Activinsights Ltd, Cambridgeshire, UK) on the wrist, which was returned (for data download) and replaced biweekly or every 4 weeks. Continuous raw data were recorded at 50 or 20 Hz and converted into daily summaries.^26,27^ Outcomes that were measured in seconds per day were converted into minutes to allow for more intuitive interpretation. Moderate and vigorous activities in minutes per day were coded into one extra variable [duration of moderate-to-vigorous intensity physical activity per day (MVPA)]. Active intensity per day and M6 intensity (intensity of most active 6 minutes) were both multiplied by 1000 since these outcomes were measured in relatively small units. Mean values were calculated for each 28-day period.

**Table 1:** Definition of the behavioural digital biomarkers

Behavioural digital biomarker	Definition
Inactive duration	The total duration of inactive events (<40 mg average acceleration magnitude) excluding sleep in a 24-h period.
Active duration	The total duration of light, moderate, and vigorous events (>40 mg average acceleration magnitude) in a 24-h period.
MVPA duration	The total duration of moderate and vigorous events (>644 mg average acceleration magnitude) in a 24-h period.
Inactive event count	The number of inactive events (outside of the rest interval) in a 24-h period
Active event count	The number of active events (outside of the rest interval) in a 24-h period
Inactive event median	The median length of inactive events (outside of the rest interval) in a 24-h period
Active event median	The median length of active events (outside of the rest interval) in a 24-h period
M6 intensity	The intensity (mean acceleration magnitude) of the most active 6 min in a 24-h period.
Active intensity	The mean intensity (mean acceleration magnitude) of active events in a 24-h period.
Activity volume	The volume of activity (the sum of the products of duration and mean acceleration magnitude for every event) in a 24-h period.
Sleep onset latency	The duration of the period between the start of the rest interval and the start of the sleep interval.
Sleep interval duration	The duration of the sleep interval
Total sleep duration	The total duration of the sleep events inside the rest interval
Sleep efficiency	The percentage of time asleep within the rest interval
Wake after sleep onset (WASO) count	The number of non-sleep events (inactive and active) inside the sleep interval
Wake after sleep onset median	The median duration of non-sleep events (inactive and active) inside the sleep interval.
Time to first WASO	The duration of the first sleep event inside the rest interval
Longest sleep period	The duration of the longest sleep event inside the rest interval
Nap duration	The total duration of sleep events outside the rest interval
Sleep event number	The number of discrete sleep events and postures in the rest interval
Total steps	The total number of steps in a 24-h period
Slow steps	The total number of steps in walking events with cadences <70 steps per minute in a 24-h period.
Fast steps	The total number of steps in walking events with cadences >70 steps per minute in a 24-h period.
Mid-sleep time	The midpoint between sleep onset and sleep end
Mean cadence	The mean cadence of walking events in a 24-h period
Cadence 95	The 95th percentile of walking event cadence in a 24-h period

^a^Events are defined as periods of >10 s duration of homogeneous accelerometer as detected by the changepoint algorithm. The sleep interval is calculated from the longest combined period of sustained inactivity in the 24-h period, and the rest interval is calculated from the active events that bracket the sleep interval.

### Statistical analysis

Continuous variables were presented by median, mean, interquartile range, and standard deviation. Categorical socio-demographic and clinical variables were presented as frequencies (percentages) and compared using the χ^2^ test. Correlation coefficients were calculated between the PROMs and each of the digital biomarkers using the Pearson correlation coefficient (PCC). Multivariable Tobit linear regression analysis was used to examine the relationship between digital biomarkers and PROMs. Tobit regression takes into account the measurement limits by censoring, which is especially suitable for PROMs with a floor-and-ceiling effect. To investigate the association between digital biomarkers and PROM domains, we fit a Tobit regression on the data collected at baseline, defined as the 28 days of biomarker data after the baseline questionnaire. We used the variance inflation factor (VIF) to quantify how much the variance of a predictor variable was inflated due to correlations with other variables in the model. Variables with a VIF value exceeding the threshold were excluded from subsequent regression analysis, retaining only variables that do not exhibit high multicollinearity. Variable selection for the final multivariable adjusted model was conducted through step-wise backward selection using the Akaike information criterion, and the estimates were reported as *β*-coefficients and 95% confidence intervals (CIs). Using the fitted model parameters of the baseline models, numerical patient-reported outcome predictions were obtained using the data collected in the 28 days leading up to the 6-month follow-up. To assess the validity of these patient-reported outcome predictions, we generated observed-vs.-predicted scatterplots and evaluated correlations by computing PCCs, Lin's concordance correlation coefficient (CCC), and Bland–Altman plot analysis. In addition, we calculated the median absolute difference (MAD) between the observed and predicted scores. A two-sided *P*-value <0.05 was considered significant. Statistical analyses were performed using the R statistical software (version 3.6.2, R Core Team), and correlation heatmaps were created using Python (version 3.6.7).^28^

## Results

**Figure 1:**
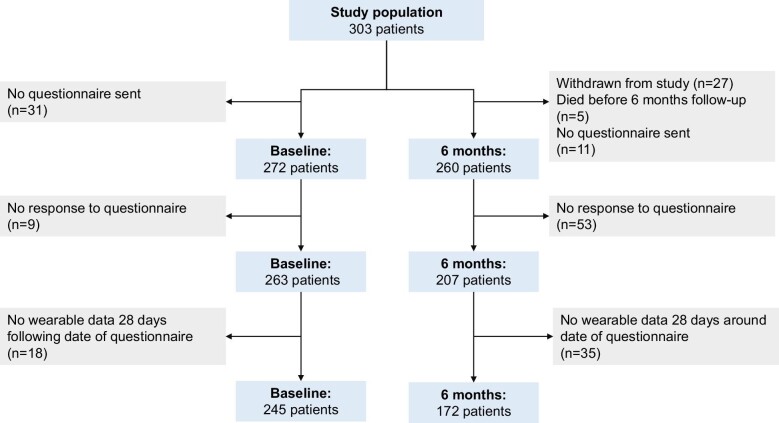
Flowchart of patient inclusion.

A total of 303 patients were included in this study [mean age 62.9 ± 10.9 years, 246 (81.2%) male, 146 (48.2%) with ischaemic cardiomyopathy, 159 (52.5%) with heart failure with reduced ejection fraction (HFrEF), 243 (80.2%) with beta-blocker treatment, 213 (70.3%) with a secondary prevention ICD, and 58 (19.1%) with CRT-D]. The baseline characteristics are displayed in *Table 2*, and *Figure 1* shows the patient-inclusion process. In our study cohort, we observed a notable gender imbalance, with males comprising 81.2% of the participants. In total, 263 patients had completed the baseline questionnaire and 207 patients completed the questionnaire at 6 months. Of the total, five patients died during follow-up, and 27 withdrew from the study during follow-up.

**Table 2:** Baseline characteristics

	Study population (*n* = 303)
Age, years (SD)	62.9 (10.9)
Male, yes (%)	246 (81.2)
Secondary prevention ICD indication,^a^ *n* (%)	213 (70.3)
Body mass index, kg/m^2^ (SD)	28.1 (6.0)
Smoking, *n* (%)	
Never	106 (35.0)
Previous	118 (38.9)
Active	35 (11.6)
Heart disease, *n* (%)	
Ischaemic	146 (48.2)
Dilated	47 (15.5)
Hypertrophic	11 (3.6)
Other	69 (22.8)
Cardiovascular history, *n* (%)	
Myocardial infarction	109 (36.0)
PCI	99 (32.7)
CABG	55 (18.2)
Heart failure (HFrEF)	159 (52.5)
Diabetes mellitus	50 (16.5)
Hypertension	157 (51.8)
Hypercholesterolemia	121 (39.9)
Cerebral vascular event	32 (10.6)
Atrial fibrillation	106 (35.0)
Medication, *n* (%)	
ACE inhibitor	122 (40.3)
Angiotensin receptor blocker	73 (24.1)
Loop diuretics	101 (33.3)
Beta-blocker	243 (80.2)
Calcium channel blockers	49 (16.2)
Lipid lowering drugs	193 (63.7)
Anti-arrhythmic class I	9 (3.0)
Anti-arrhythmic class III	50 (16.5)
Implanted device, *n* (%)	
Single-chamber	176 (58.1)
Dual-chamber	67 (22.1)
CRT-D	58 (19.1)

ACE, angiotensin-converting enzyme; CABG, coronary artery bypass grafting; CRT, cardiac resynchronization therapy; NOAC, non-vitamin K antagonist oral anticoagulant; PCI, percutaneous coronary intervention.

^a^Implantable cardioverter defibrillator (ICD) placement with the intention of preventing SCD in a patient who has had a sustained ventricular tachycardia or ventricular fibrillation.

### Descriptive analysis of the patient-reported outcomes and digital biomarkers during follow-up

**Figure 2:**
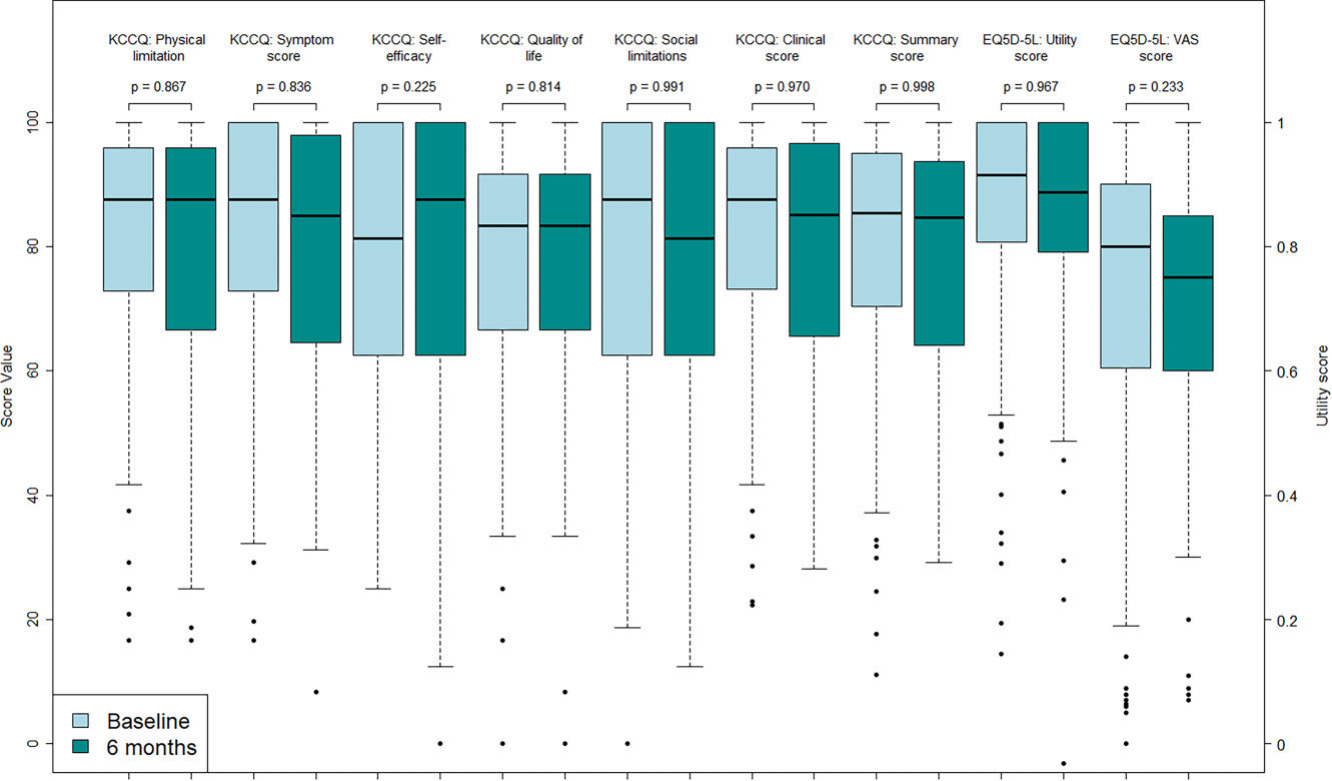
Boxplots of the patient-reported outcomes at baseline and 6-month follow-up.

The median scores for each of the KCCQ domains and the EQ5D-5L utility score at baseline and 6-month follow-up are displayed in *Figure 2* and *Table 3*. At the population level, no significant change in any of the scores was observed between baseline and 6-month follow-up. Supplementary material online, *Figure S1* illustrates the changes in PROMs, specifically the proportions of patients who experienced improvement or decline, during the 6-month follow-up period compared with their baseline PROM scores. A total of 28 715 days of wearable data were collected within the first 6 month period of the 263 patients who responded to the PROM at baseline. In the 28 day period following the baseline PROMs, a total of 6030 daily summaries of digital biomarkers were available {median of 26 [interquartile range (IQR) 24, 27] days per patient}. In the 28 days leading up to the 6-month follow-up PROM, 3870 days of wearable data were collected [24 (IQR 17, 27) days per patient]. No significant changes in digital biomarkers were observed over time, except for an increase in sleep interval duration. Mean values for the digital biomarkers are displayed in Supplementary material online, *Table S2*.

**Table 3:** Patient-reported outcomes at baseline and at 6-month follow-up

PROM	Domain	Baseline (*n* = 263)	6 months (*n* = 207)	*P*-value
Kansas City Cardiomyopathy Questionnaire	Summary, median (IQR)	84.90 [69.3, 95.3]	85.42 [66.2, 93.8]	0.998
	Clinical, median (IQR)	86.46 [72.9, 96.4]	86.46 [69.8, 96.8]	0.970
	Symptoms, median (IQR)	85.42 [70.8, 97.9]	86.46 [66.7, 97.9]	0.836
	Self-efficacy, median (IQR)	75.00 [62.5, 100.0]	87.50 [62.5, 100.0]	0.225
	Quality of life, median (IQR)	83.33 [66.7, 91.7]	83.33 [66.7, 100.0]	0.814
	Social limitation, median (IQR)	87.50 [62.5, 100.0]	83.33 [62.5, 100.0]	0.991
	Physical limitation, median (IQR)	87.50 [75.00, 95.83]	87.50 [70.0, 95.83]	0.867
EQ5D-5L	Utility score, median (IQR)	0.91 [0.81, 1.0]	0.91 [0.8, 1.0]	0.967
	VAS score, median (IQR)	80.00 [64.0, 90.0]	75.00 [60.0, 89.0]	0.233

IQR, interquartile range.

### Correlations between biomarkers and patient-reported outcomes

**Figure 3:**
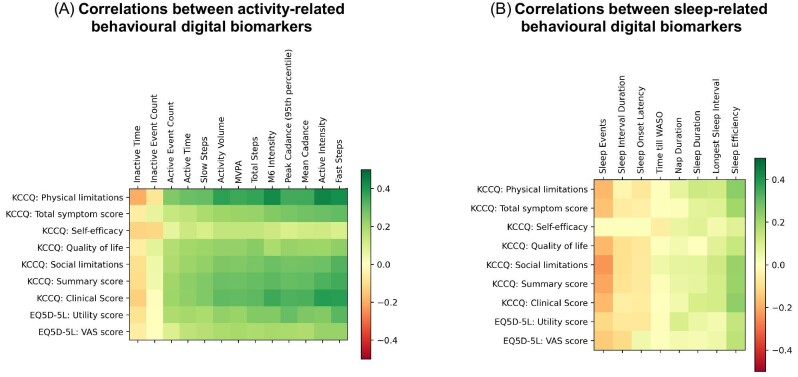
Correlation heatmap between patient-reported outcome measures (PROMs) and behavioural digital biomarkers, stratified by activity-related (*A*) and sleep-related metrics (*B*).

We examined the correlations between behavioural digital biomarkers, KCCQ domains, and the EQ5D-5L health-related quality of life scores, integrating data collected at baseline and at 6-month follow-up. *Figure 3* shows the heatmap of each pair of PROM and behavioural digital biomarker, numeric values of the correlation coefficients are provided in Supplementary material online, *Tables S3* and *S4*. Significant correlations were observed, with the strongest associations found between patient-reported physical limitations and the active intensity (*r* = 0.453; *P* < 0.001), intensity of most active 6 min (*r* = 0.438; *P* < 0.001), and number of fast steps (*r* = 0.421; *P* < 0.001). Correlations coefficients for the KCCQ summary score, clinical score, subdomains of social limitations, and total symptom score demonstrated stronger associations between behavioural biomarkers compared with health-related quality of life and self-efficacy. In terms of sleep behaviour, correlations between sleep efficiency and the KCCQ summary and clinical score (*r* = 0.211; *P* < 0.001 and *r* = 0.232; *P* < 0.001, respectively), physical and social limitations (*r* = 0.242; *P* < 0.001 and *r* = 0.205; *P* < 0.001, respectively), and the symptom score (*r* = 0.202; *P* < 0.001) were strongest.

### Multivariable Tobit models

Associations between behavioural digital biomarkers and PROMs were then examined through stepwise regression analyses, using data collected over a 28-day period following the completion of the baseline PROM. The effect sizes and 95% CIs are presented in the forest plots, shown in Supplementary material online, *Figure S2*. The KCCQ summary score was significantly associated with the number of inactive events (*β* = 0.04, *P* = 0.001), median duration of active events (*β* = −0.40, *P* = 0.013), activity volume (*β* = 0.01, *P* < 0.001), number of fast steps (*β* = −0.28, *P* = 0.016), and sleep efficiency (*β* = 0.58, *P* < 0.001). The EQ5D-5L utility score for health-related quality of life was associated with active event duration (*β* = −0.003, *P* = 0.018), active volume (*β* = 0.00004, *P* = 0.023), peak cadence (*β* = 0.004, *P* < 0.001), number of fast steps (per 100 steps, *β* = −0.002, *P* = 0.30), time to WASO (*β* = −0.022, *P* = 0.001), and sleep event count (*β* = −0.001, *P* = 0.003). Clinical variables, including age, sex, atrial fibrillation, chronic kidney disease, and the use of loop diuretics, demonstrated significant associations with multiple PROMs.

### Prospective model validation

**Figure 4:**
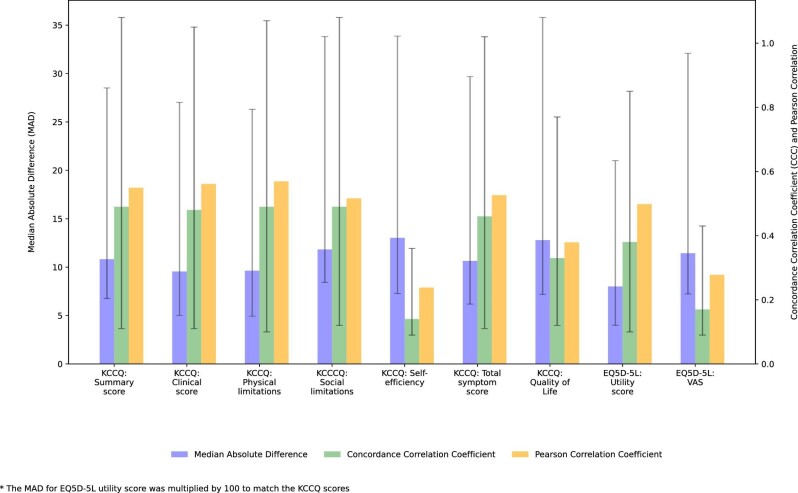
Validation of the multivariable Tobit regression model for prediction of each patient-reported outcome.

**Figure 5:**
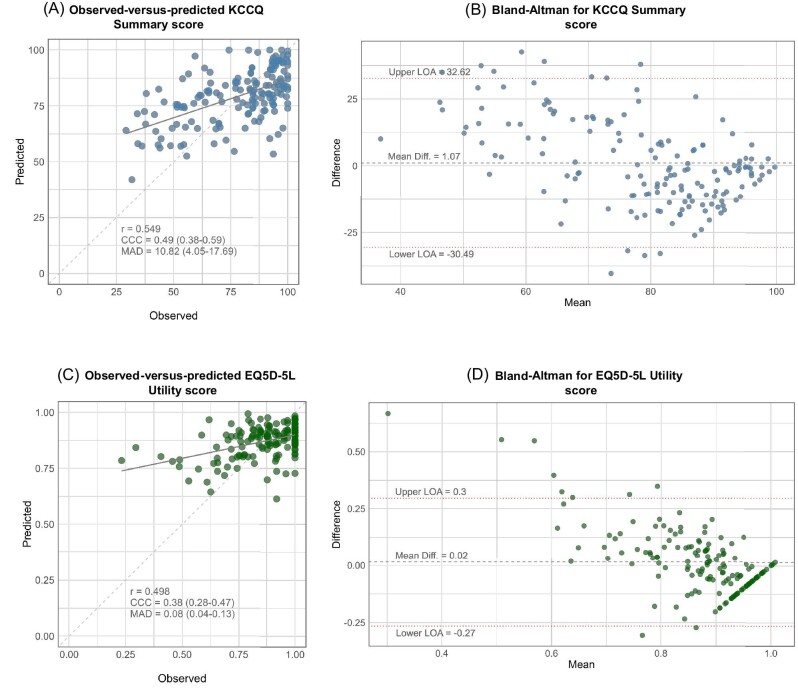
Observed-vs.-predicted scatterplot and Bland–Altman plot for the Kansas City Cardiomyopathy Questionnaire summary score (*A*–*B*) and the EuroQoL 5-Dimensions 5-Levels utility score (*C*–*D*).

To evaluate the accuracy of the models, we used prospectively collected data over a 28-day period leading up to the PROM at 6-month follow-up to numerically predict the outcome. The MAD, CCC, and PCC for each of PROMs are displayed in *Figure 4*. With respect to the KCCQ summary score, the MAD between the observed and predicted score was 10.8 (IQR 4.1, 17.7), with a PCC of 0.549 and a CCC of 0.49 (95% CI 0.38, 0.59) (*Figure 5A*). *Figure 5B* shows the Bland–Altman plot, the average mean difference between measures was low, but limits of agreement (LOA) were wide [mean difference 1.07, 95% LOA (range −30.49 to 32.62)]. The MAD for EQ5D-5L utility score was 0.08 (IQR 0.04, 0.13), with a PCC of 0.498 and a CCC of 0.38 (95% CI 0.28, 0.47) (*Figure 5C*). The mean difference was 0.02 (LOA range −0.27 to 0.33) (*Figure 5D*). For both the KCCQ summary score and the EQ5D-5L utility score, the errors were non-normally distributed with an overestimation at low values. The performance of the model to estimate patient-reported self-efficacy was poor with a PCC of 0.238 and a CCC of 0.14 (95% CI 0.05, 0.22). Supplementary material online, *Figure S3* shows the scatterplots for observed-vs.-predicted and Bland–Altman plots for each PROM.

## Discussion

The growing availability of wearable technology offers a wealth of personalized data that provide insights into behavioural patterns in real-world settings. We sought to uncover the potential of wearables to monitor PROMs by investigating associations between longitudinal behavioural digital biomarkers and validated PROMs, and by exploring the capacity of these biomarkers to predict the same. Using data from a prospective study, we fitted regression models on baseline data to probe factors that predict PROMs, and as a proof of concept, we applied these models to biomarker measurements collected over time. First, we observed significant associations between behavioural digital biomarkers and PROMs. Second, there were moderate correlations between the observed and predicted values derived from the regression models, in particular for PROMs that assess physical and social limitations, frequency and severity of symptoms, and quality of life. Notably, biomarkers linked to activity intensity and volume were consistently associated with PROMs. These findings imply that a panel of objective behavioural digital biomarker measurements can serve as a robust and reliable proxy for subjective PROMs, facilitating continuous monitoring of PROMs. Integration of a prediction model that provides personalized patient-reported outcome predictions and alarms clinicians if a meaningful change is expected could enable clinicians to proactively approach individuals to collect PROM data and tailor patient care.

Globally, there is growing momentum to incorporate PROMs into routine clinical practice. PROMs provide important indicators of treatment efficacy not captured by objective markers or clinical assessment, and play a crucial role in health technology assessments.^29^ PROMs, being subjective, can be influenced by internal factors (mood, expectations) and external factors (treatment context, socio-economic status), leading to outcome fluctuations.^12^ Collecting measurements at multiple time points helps mitigate this variability; however, frequent administration of PROMs comes with an increase in respondent burden, potentially resulting in higher respondent burden, non-response bias, and missing data.^13,29^ Also, considering the variation of respondent burden across populations, influenced by factors such as socio-economic status, literacy levels, and cognitive impairment, reliance on PROMs without other measures could perpetuate social inequities and exacerbate disparities.^30^ From the perspective of healthcare providers, the influx of PROM data poses challenges as it necessitates clinician oversight and resources for interpretation, action, and discussion with patients.^31^ To address these challenges, we explored the potential for personalized, real-time estimation of PROMs through a panel of digital biomarkers collected in a free-living environment. Our findings indicated that by leveraging behavioural digital biomarkers, we were able to approximate PROMs with moderate correlations to the actual scores. However, for the clinical adoption of such a tool, it is imperative that the model can maintain prediction errors below the MCID threshold. Furthermore, our findings consistently highlight the significant impact of clinical factors on the PROM scores. Notably, factors such as the presence of atrial fibrillation, use of loop diuretics, CRT, age, and gender have been shown to be significantly associated with PROM scores. This underscores the need for incorporation of both wearable-derived data and clinical variables, as they both contribute substantially to the outcomes, and the need for further exploration of potential predictors.

**Figure 6:**
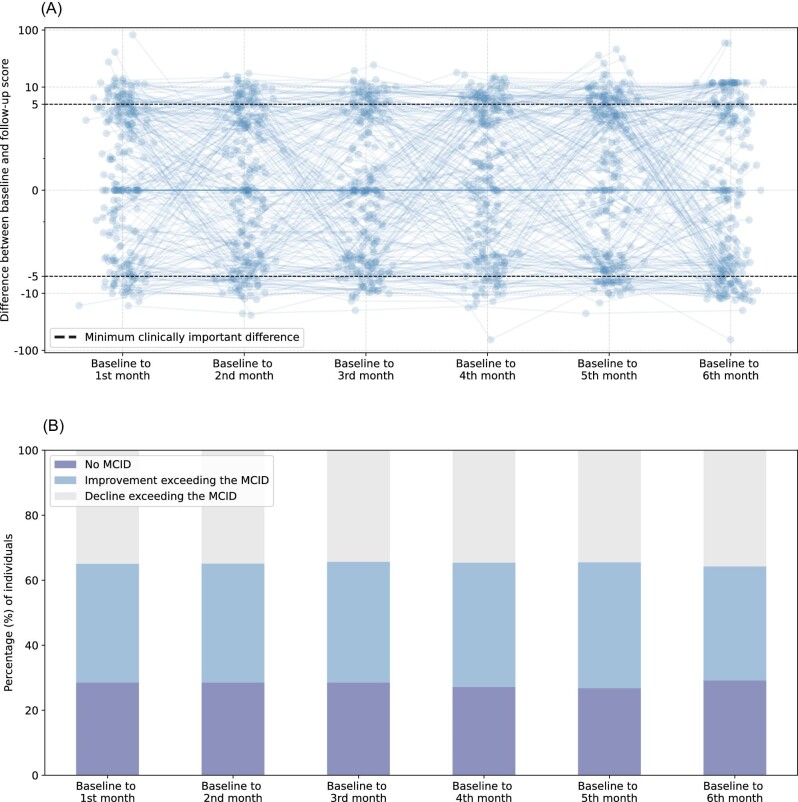
KCCQ summary score change over time relative to baseline values (*A*) and proportions of improvement/decline (*B*). (*A*) Each blue dot represents the predicted KCCQ summary scores for a patient relative to baseline values. Tobit regression models were developed using the data collected during the 28 days following baseline, and used to predict the KCCQ summary scores over each subsequent 28-day interval. Dashed lines denote the clinically significant threshold, crossing of this line indicates a potential clinically relevant improvement or decline in the PROM. (*B*) The percentage of patients where a clinically significant change in the KCCQ summary score is predicted, relative to their baseline value. KCCQ, Kansas City Cardiomyopathy Questionnaire; MCID, minimum clinically important difference; PROM, patient-reported outcome measure.

Setting a threshold on the MCID, *Figure 6* demonstrates how patient-reported outcome prediction models can utilize digital biomarkers to identify individuals at risk of experiencing a clinically relevant change in PROM. Leveraging this approach may enable clinicians to personalize the timing of PROMs to specifically target individuals with an actionable PROM score.^32^

Previously, studies have evaluated the associations between behavioural digital biomarkers and PROMs in patients with heart failure, atrial fibrillation, and ICD carriers.^14–20^ To our knowledge, this is the first study to provide a framework for real-time monitoring of PROMs through wearable-collected behavioural digital biomarkers. This study extends the findings from the study by Werhahn *et al*., who developed a remote monitoring platform for heart failure patients that relied on commercially available wearable devices to correlate measures of daily step counts to PROMs.^19^ In contrast to this study, by using research-graded wearable accelerometers and dedicated algorithms to process the raw accelerometry data, we were able to measure a more comprehensive set of variables that quantifies multiple domains of physical behaviour, including information on duration, intensity, and volume of physical activity and sleep behaviour. Interestingly, although extensively used in prior studies, our findings have demonstrated that the number of daily steps and the duration in MVPA were not associated with PROMs.^33,34^ In addition to metrics related to activity intensity (including peak and mean cadence, daily fast steps), activity volume, and duration of activity, sleep efficiency was consistently associated with each of the PROMs, which emphasizes the value of a multidimensional approach to physical behaviour.^35^ In our models, we see negative coefficients for active event duration and fast steps, despite these having positive correlations with improved PROM scores generally. This is possibly linked to the patterning and fragmentation of within-day behaviour for which we did not have biomarkers. Further work in this area could provide insights to improve the predictive power of these types of models.

Prior studies have predominantly relied on 7-day monitoring periods to collect wearable-derived data. However, ∼40% of the variance in physical behaviour during a 7-day monitoring period can be explained by random error caused by behavioural variability, and as a result may not be representative of an individual's physical behaviour over a longer period of time.^36^ By using data from a prospective study, we were able to use data collected over a 6-month period. Furthermore, significant variations were observed in the strength of correlations and associations between behavioural digital biomarkers and various PROMs. In contrast to performance on patient-reported physical and social limitations, severity and frequency of symptoms, and quality of life, we found the predictive ability of behavioural biomarkers for the patient-reported self-efficacy to be poor. Therefore, it is important that future studies further explore the specific PROM domains that can be effectively approximated using behavioural digital biomarkers.

It is important to consider the relatively small sample size in this study, as the generalizability of the findings to a broader population and the statistical power of the analyses may be compromised. Especially, the high prevalence of male subjects in our patient cohort deserves attention. The reason for the high prevalence of males in our study cohort may be multifactorial. First, this gender distribution reflects a known substantially higher proportion of male ICD carriers, in particular patients with a primary prevention ICD indication.^37^ Second, while our study did not specifically investigate the underlying reasons for this gender imbalance, this is in line with underrepresentation of women in clinical trials.^38^ Future research should explore the potential implications of the gender imbalance in ICD trials and the adoption of digital tools to ensure equitable healthcare delivery and research participation. Related to this, it is important that future studies investigate the applicability of these findings in a general population and other patient subpopulations.

### Limitations

There are a few limitations that should be acknowledged. First, a substantial percentage of missing data was observed due to various factors, including non-response to PROMs, non-adherence to the wearable device, and participant dropout. The decentralized nature of the study, where participants were instructed to wear wearable devices continuously for 365 days, including both day- and night-time periods, presents challenges for patient adherence to wearing the devices, compliance with completing PROMs, and increases the risk of participant dropout. These instances of missing data can introduce selection bias and may affect the generalizability of the study findings. Second, in this study, we did not account for clinical disease progression; however, prior research has suggested that associations between behavioural digital biomarkers and PROMs in patients whose physical activity levels decline to very low values may be altered.^15^ Therefore, future studies are warranted to simultaneously examine the effect of clinical worsening on both behavioural biomarkers and PROMs. Third, the KCCQ was developed and validated for patients with heart failure with reduced or preserved ejection fraction. Given that a substantial proportion of our patient cohort was not diagnosed with HFrEF at study enrolment, the KCCQ may not be appropriate for the entire patient population.

## Conclusion

Digital biomarkers that reflect physical behaviour can be used to predict and monitor patient-reported physical and social limitations, severity and frequency of symptoms, and health-related quality of life. This may be the next step towards personalized, real-time monitoring of PROMs in a remote setting with systems that incorporate wearable accelerometers. Future studies are warranted to assess the validity of these results in different patient populations, during longer follow-up and in larger sample sizes.

## Supplementary Material

qcad069_Supplemental_File

## Data Availability

The data underlying this article will be shared on reasonable request to the corresponding author.

## Notes

**Conflict of interest:** R.E.K. reports consultancy fees and research grants from Boston Scientific, Medtronic, and Abbott and has stock options from AtaCor Medical Inc. F.V.Y.T. has received grants or contracts from the Dutch Research Council (NWO) and Amsterdam Cardiovascular Sciences, and received payment or honoraria from Boston Scientific and Abbott (no personal financial gain). S.Z.D. reports consultancy fees and research grants from Acesion Pharma and Cortrium, and has received payment or honoraria from Bristol Myers Squibb, Pfizer, and Bayer. P.K.J. reports consultancy fees and research grants from Abbott and Medtronic, payment or honoraria from Abbott and Medtronic, and support for attending meetings and/or travel from Abbott and Medtronic. J.H.S. reports grants or contracts from Medtronic (paid to institution), payment or honoraria from Medtronic, support for attending meetings and/or travel from Abbott and Medtronic, participation on Medtronic Advisory Board, and stocks or stock options from Vital Beats. J.L. reports having stock or stock options from Activinsights Ltd. T.O.A. reports having stock or stock options from Vital Beats. D.M.F. reports support for attending meetings and/or travel from Boston Scientific. M.Z.H.K., N.R., H.L.T., and C.M. have nothing to declare.
