# Supplementary material for: Behavioural digital biomarkers enable real-time monitoring of patient-reported outcomes: a substudy of the multicentre, prospective observational SafeHeart study
Source: Eur Heart J Qual Care Clin Outcomes. 2023 Dec 6;10(6):531–42. doi: 10.1093/ehjqcco/qcad069 (PMC11873796; doi:10.1093/ehjqcco/qcad069)
Supplement: qcad069_Supplemental_File [file qcad069_supplemental_file.docx]

**SUPPLEMENTARY** **MATERIAL**

**Supplemetary Figures**

Supplementary Figure 1. Alluvial plot of the baseline and 6 months response to the patient-reported outcome

Supplementary Figure 2. Forest plot of regression models for each patient-reported outcomes

Supplementary Figure 3. Scatterplots and Bland-Altman plots of the observed-versus-predicted patient-reported outcome measures at 6 months follow-up for the KCCQ subdomains and EQ5D-5L VAS score

**Supplemetary Tables**

Supplementary Table 1. Baseline characteristics of the responders to the baseline questionnaire

Supplementary Table 2. Wearable-derived behavioural digital biomarkers at baseline and at 6 months follow-up (complete cases)

Supplementary Table 3. Pearson correlation coefficients for activity-related digital biomarkers and patient-reported outcomes

Supplementary Table 4. Pearson correlation coefficients for sleep-related digital biomarkers and patient-reported outcomes

**Supplementary Figure 1.** Alluvial plot of the baseline and 6 months response to the patient-reported outcome measure**
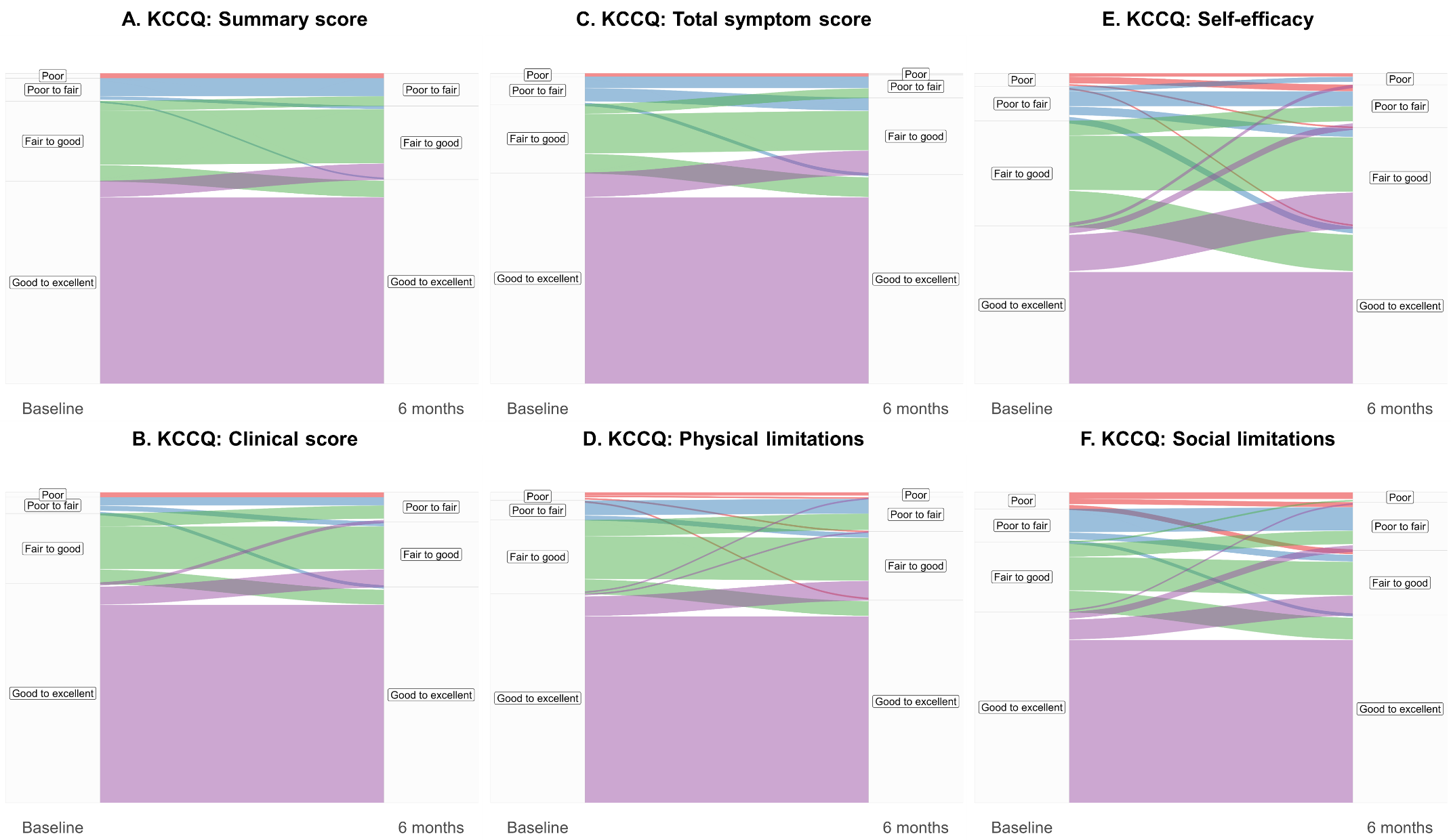
**

**
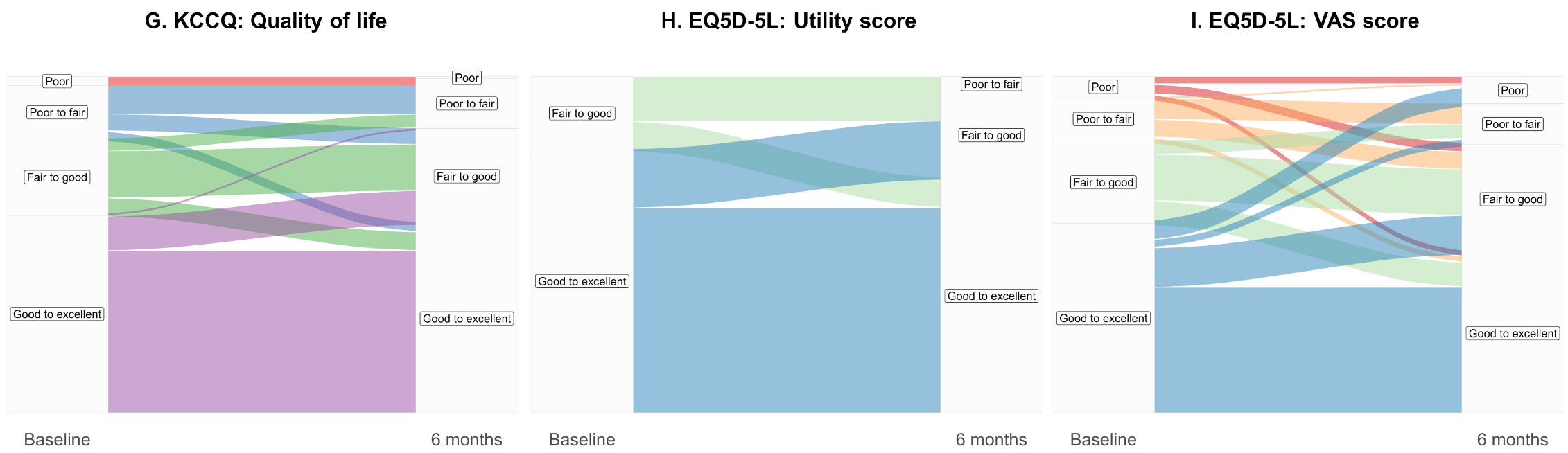
**

**
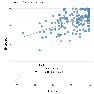

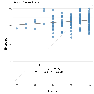

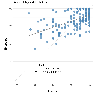

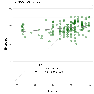

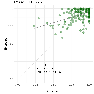
Supplementary Figure 2.** Forest plot of regression models for each patient-reported outcome measure. Total step counts, slow steps and fast steps in 100-step units.
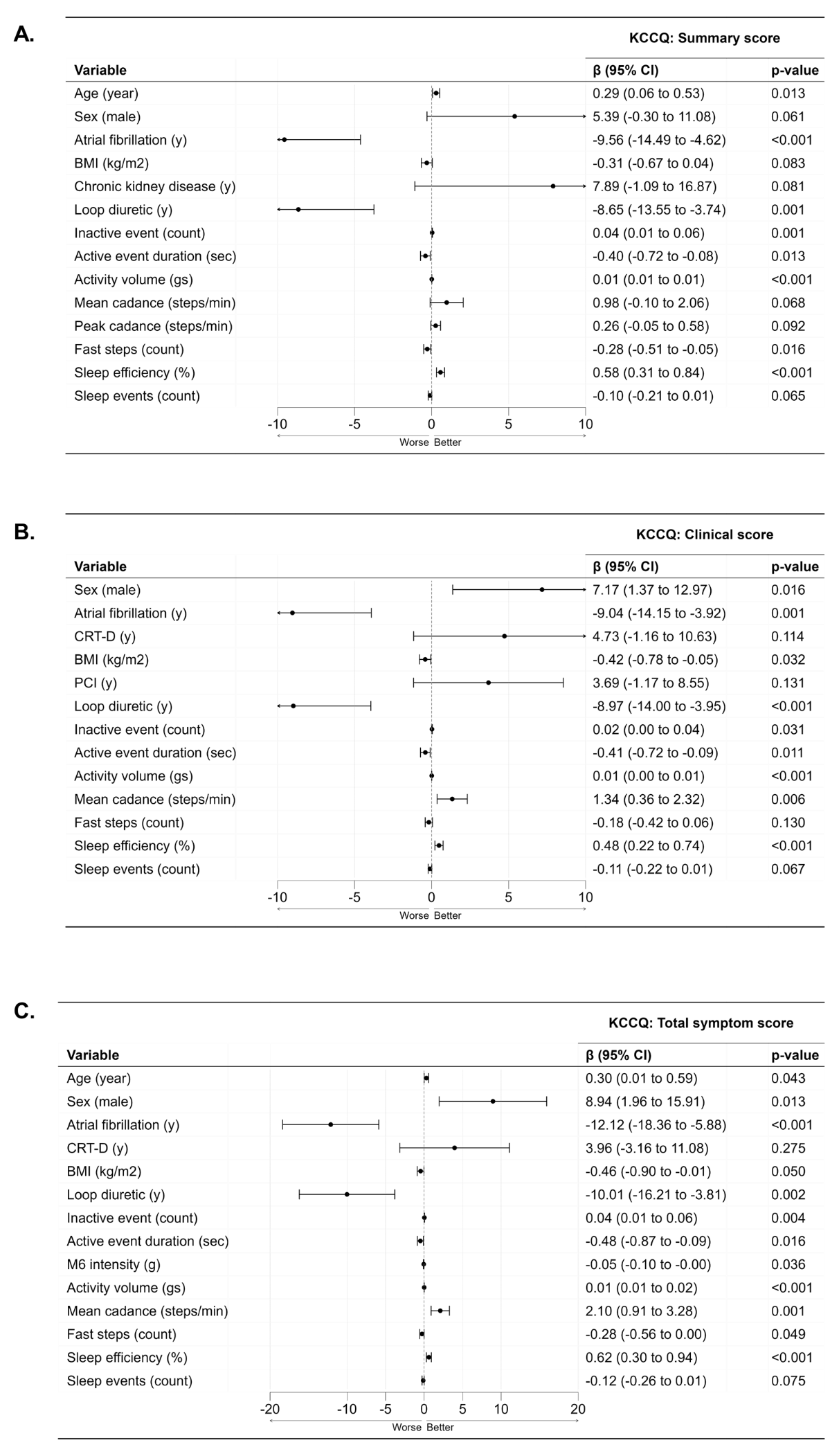

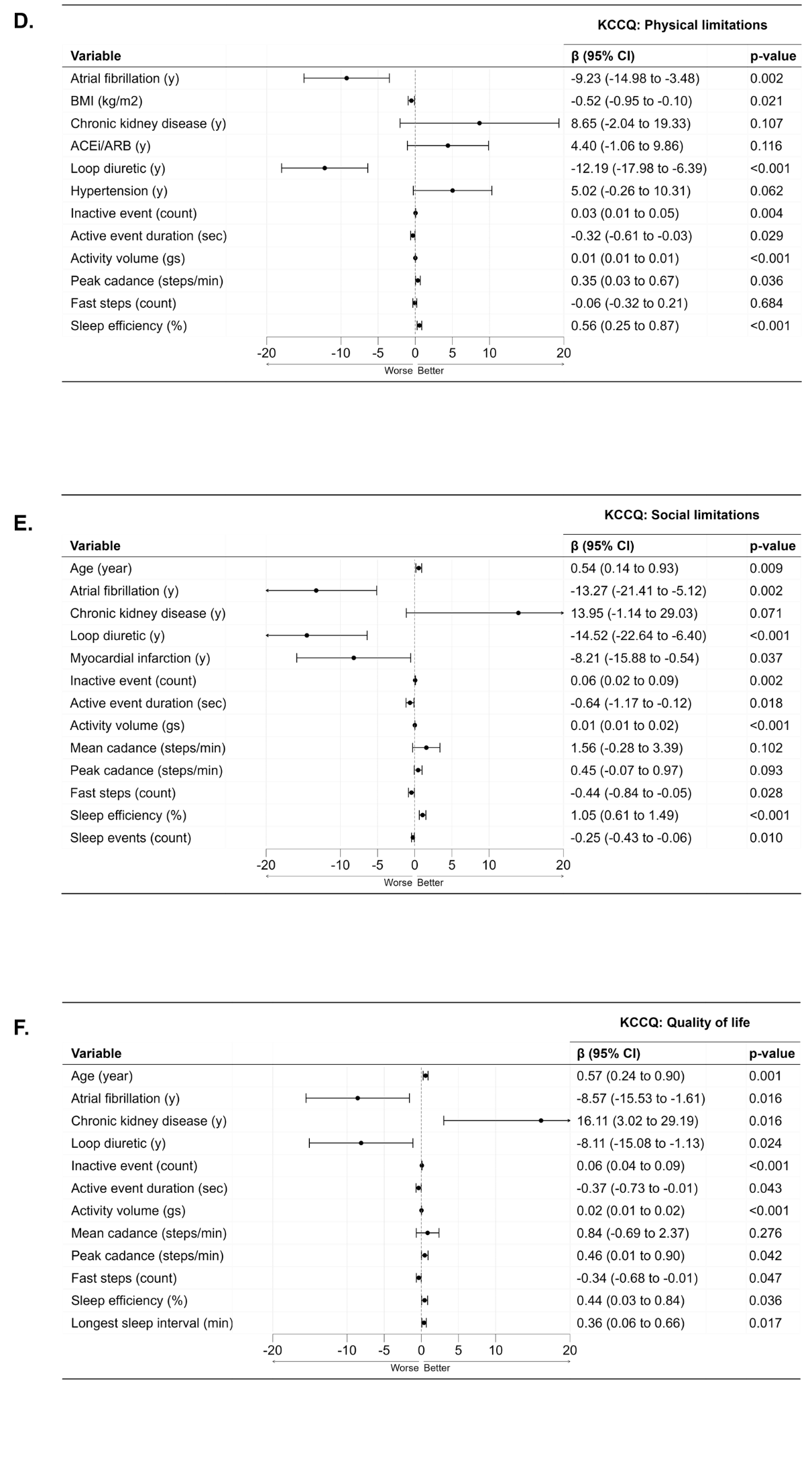
**
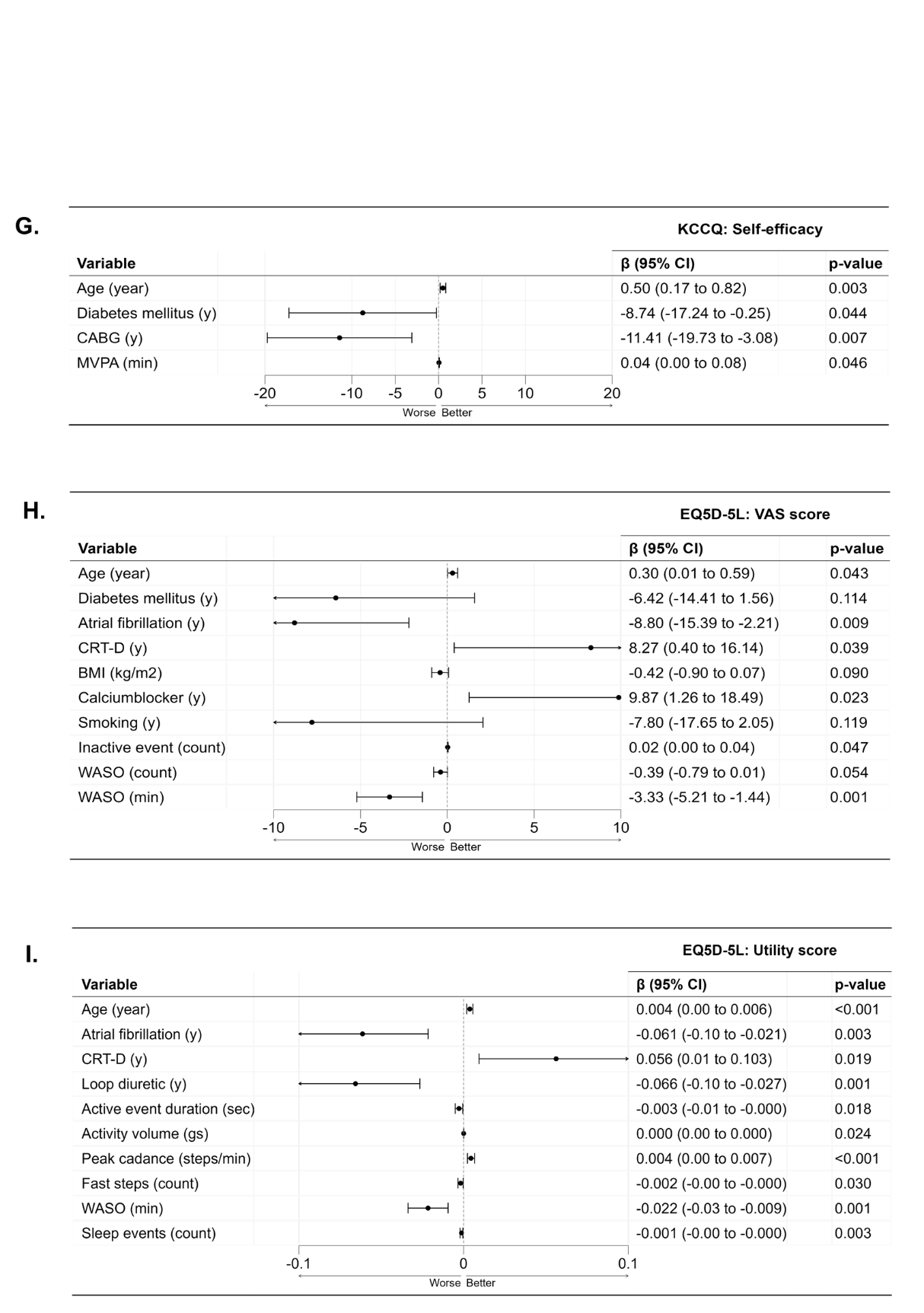
Supplementary Figure 3.** Scatterplots and Bland-Altman plots of the observed-versus-predicted patient-reported outcome meausures at 6 months follow-up for the KCCQ subdomains and EQ5D-5L VAS score


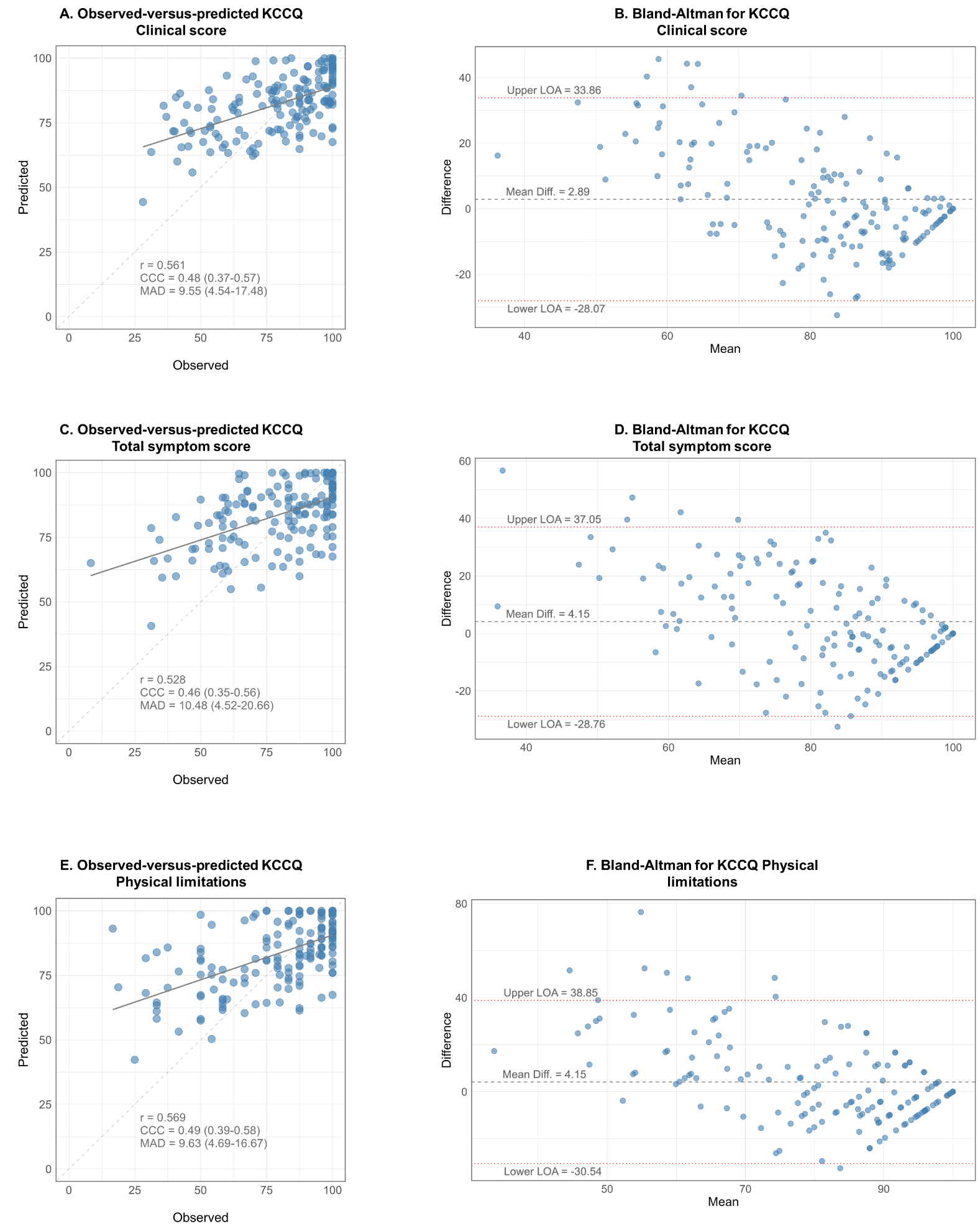

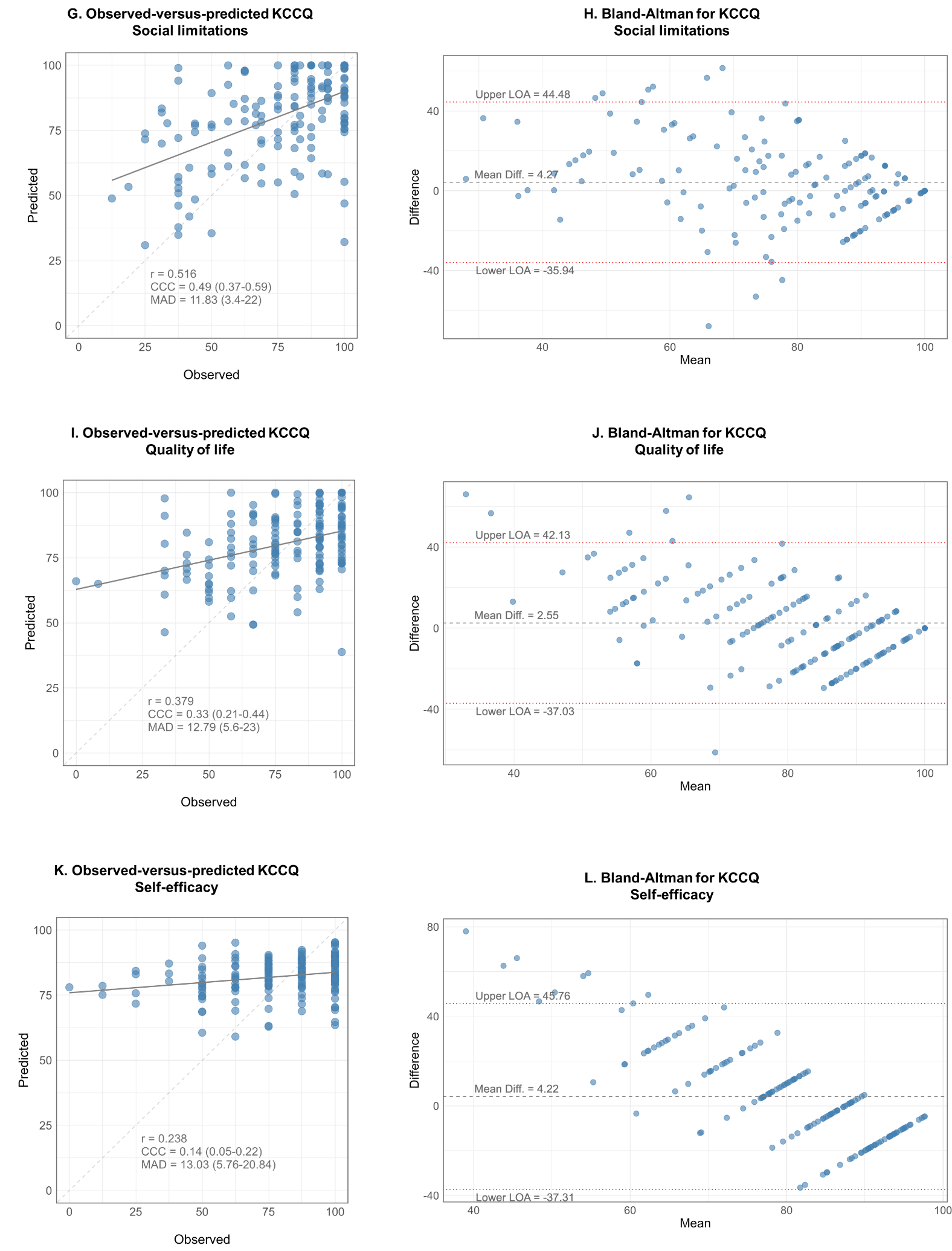


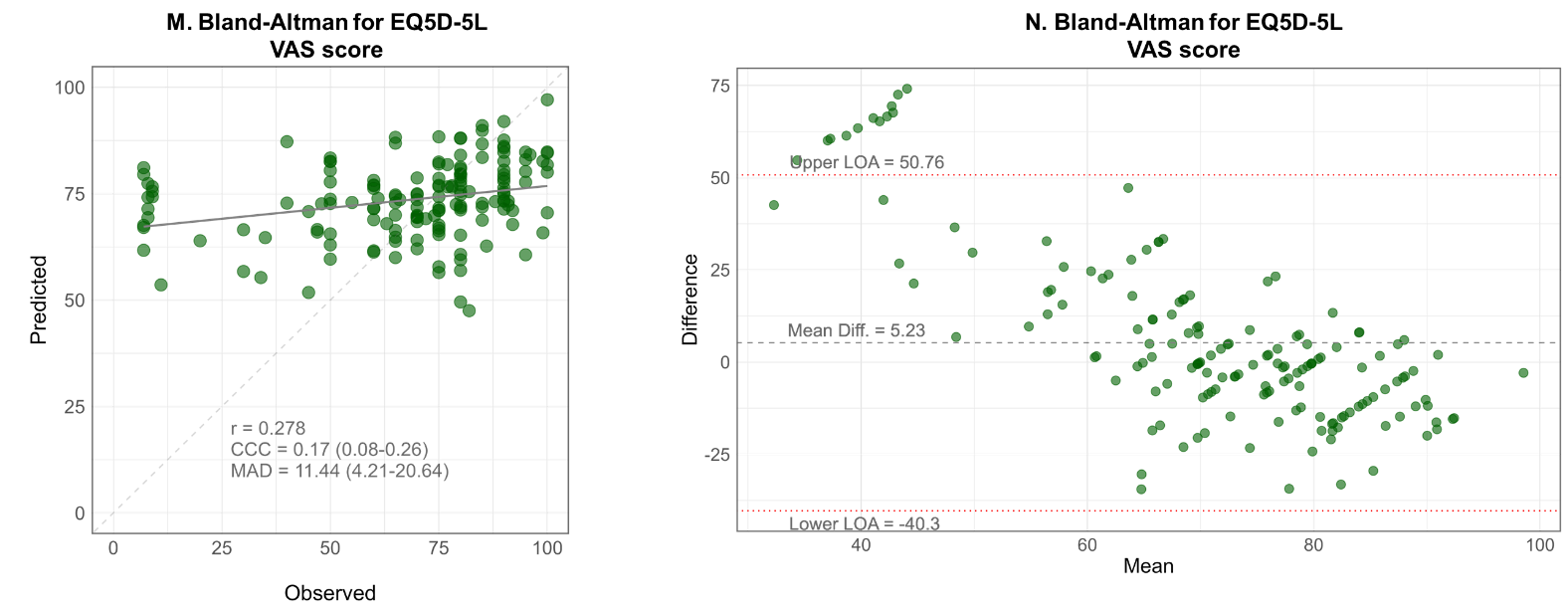
**Supplementary Table 1.** Baseline characteristics of the responders to the baseline questionnaire

|  | | *Responders (n=245)* |
| --- | --- | --- |
| Age, years (SD) | | 63.60 (9.44) |
| Male, yes (%) | | 135 (82.3) |
| Secondary prevention, yes (%) | | 111 (67.7) |
| Body mass index, (SD) | | 27.87 (5.01) |
| Smoking, yes (%) | |  |
|  | Never | 55 (33.5) |
|  | Previous | 62 (37.8) |
|  | Active | 19 (11.6) |
| *Cardiomyopathy, yes (%)* | |  |
|  | Ischaemic | 77 (47.0) |
|  | Dilated | 30 (18.3) |
|  | Hypertrophic | 6 (3.7) |
|  | Other | 63 (25.7) |
| *Cardiovascular history, yes (%)* | |  |
|  | Myocardial infarction | 52 (31.7) |
|  | PCI | 52 (31.7) |
|  | CABG | 31 (18.9) |
|  | Heart failure (HFrEF) | 91 (55.5) |
|  | Diabetes mellitus |  |
|  | Hypertension | 81 (49.4) |
|  | Hypercholesterolemia | 61 (37.2) |
|  | Peripheral artery disease | 7 (4.3) |
|  | Cerebral vascular accident | 15 (9.1) |
|  | Atrial fibrillation | 53 (32.3) |
| *Medication, yes (%)* | |  |
|  | ACE inhibitor | 58 (35.4) |
|  | Angiotensin receptor locker | 44 (26.8) |
|  | Loop diuretics | 55 (33.5) |
|  | Beta-blocker | 133 (81.1) |
|  | Calcium channel blockers | 22 (13.4) |
|  | Nitrates | 23 (14.0) |
|  | NOAC | 38 (23.2) |
|  | Vitamin K antagonist | 22 (13.4) |
|  | Lipid lowering drugs | 103 (62.8) |
| *Implanted device, yes (%)* | |  |
|  | Single-chamber | 98 (59.8) |
|  | Dual-chamber | 31 (18.9) |
|  | CRT-D | 34 (20.7) |
| ACE: Angiotensin-Converting Enzyme, CABG: Coronary Artery Bypass Grafting, CRT: Cardiac Resynchronisation Therapy, NOAC: Non-vitamin K Antagonist Oral Anticoagulant, PCI: Percutaneous Coronary Intervention | | |

**Supplementary Table 2.** Wearable-derived daily behavioural digital biomarkers at baseline and at 6 months follow-up (complete cases)

|  | Baseline | 6 months | P-value |
| --- | --- | --- | --- |
| Inactive duration (min) | 733.96 (123.80) | 732.38 (121.94) | 0.909 |
| Active duration (min) | 289.27 (97.11) | 294.53 (103.91) | 0.640 |
| Light duration (min) | 149.23 (43.17) | 155.13 (50.99) | 0.265 |
| Moderate duration (min) | 138.28 (77.52) | 138.19 (78.25) | 0.992 |
| Vigorous duration (min) | 1.76 (5.10) | 1.21 (3.09) | 0.247 |
| Inactive events (count) | 783.86 (165.81) | 785.05 (161.21) | 0.948 |
| Active events (count) | 221.76 (52.66) | 222.94 (55.07) | 0.845 |
| Inactive event median duration (min) | 0.63 (0.05) | 0.63 (0.06) | 0.650 |
| Active event median duration (min) | 0.63 (0.18) | 0.63 (0.20) | 0.903 |
| M6 intensity (m*g*) | 240.46 (81.35) | 234.59 (76.08) | 0.505 |
| Active intensity (m*g*) | 122.56 (19.66) | 120.56 (17.52) | 0.337 |
| Activity volume (gs) | 2198.98 (970.56) | 2200.63 (953.15) | 0.988 |
| Sleep onset latency (min) | 7.58 (4.67) | 7.36 (4.87) | 0.680 |
| Sleep interval duration (min) | 540.83 (111.87) | 569.61 (142.73) | 0.046 |
| Total sleep duration (min) | 322.39 (85.10) | 324.04 (86.88) | 0.864 |
| Sleep efficiency (%) | 60.15 (10.01) | 57.98 (10.39) | 0.059 |
| WASO (count) | 23.79 (7.01) | 24.98 (8.33) | 0.168 |
| WASO median (min) | 5.63 (1.72) | 5.66 (1.72) | 0.880 |
| Time to first WASO (min) | 9.74 (4.89) | 9.16 (5.12) | 0.301 |
| Longest sleep period (min) | 51.52 (12.23) | 52.02 (12.13) | 0.712 |
| Naps duration (min) | 11.16 (6.67) | 11.95 (12.89) | 0.494 |
| Sleep events (count) | 111.41 (27.23) | 109.15 (25.01) | 0.440 |
| Total steps (per 100 counts) | 121.70 (51.00) | 122.04 (51.39) | 0.952 |
| Mean cadence (steps/min) | 56.25 (4.56) | 55.53 (4.04) | 0.139 |
| Peak cadence (steps/min) | 84.89 (14.02) | 82.71 (12.79) | 0.148 |
| Slow steps (per 100 counts) | 56.34 (33.55) | 56.97 (34.21) | 0.869 |
| Fast steps (per 100 counts) | 23.86 (23.32) | 21.53 (19.64) | 0.334 |
| Midsleep time (min) | 652.77 (68.91) | 652.72 (73.98) | 0.995 |
| MVPA duration (min) | 140.03 (79.46) | 139.40 (79.17) | 0.943 |

**Supplementary Table 3.** Pearson correlation coefficients for activity-related digital biomarkers and patient-reported outcomes. No adjustment for multiple comparisons was performed.

|  | **KCCQ: Summary score** | **KCCQ: Clinical Score** | **KCCQ: Physical limitations** | **KCCQ: Quality of life** | **KCCQ: Self-efficacy** | **KCCQ: Social limitations** | **KCCQ: Total symptom** | **EQ 5D-5L Utility** | **EQ 5D-5L VAS** | |
| --- | --- | --- | --- | --- | --- | --- | --- | --- | --- | --- |
| Active intensity | 0.355* | 0.399* | 0.453* | 0.222* | 0.111* | 0.315* | 0.307* | 0.262* | 0.221* | |
| M6 intensity | 0.338* | 0.367* | 0.438* | 0.220* | 0.123* | 0.313* | 0.263* | 0.292* | 0.192* | |
| Fast steps | 0.355* | 0.383* | 0.421* | 0.248* | 0.117* | 0.334* | 0.304* | 0.310* | 0.221* | |
| Activity volume | 0.306* | 0.324* | 0.394* | 0.224* | 0.142* | 0.268* | 0.222* | 0.232* | 0.187* | |
| Total steps | 0.309* | 0.322* | 0.381* | 0.241* | 0.144* | 0.269* | 0.228* | 0.250* | 0.187* | |
| MVPA | 0.312* | 0.324* | 0.380* | 0.233* | 0.140* | 0.274* | 0.234* | 0.225* | 0.194* | |
| Mean cadence | 0.320* | 0.342* | 0.356* | 0.232* | 0.125* | 0.300* | 0.290* | 0.263* | 0.182* | |
| Peak cadence | 0.308* | 0.325* | 0.347* | 0.234* | 0.114* | 0.288* | 0.265* | 0.290* | 0.188* | |
| Slow steps | 0.264* | 0.270* | 0.319* | 0.206* | 0.099* | 0.228* | 0.194* | 0.191* | 0.167* | |
| Active duration | 0.245* | 0.251* | 0.310* | 0.197* | 0.130* | 0.209* | 0.164* | 0.205* | 0.150* | |
| Active event count | 0.199* | 0.199* | 0.240* | 0.182* | 0.055 | 0.169* | 0.139* | 0.182* | 0.088* | |
| Inactive duration | -0.130* | -0.159* | -0.228* | -0.058 | -0.127* | -0.116* | -0.075 | -0.110* | -0.058 | |
| Inactive event count | 0.033 | -0.016 | -0.088 | 0.080 | -0.109* | 0.043 | 0.058 | -0.002 | 0.004 | |
| * *P* < 0.05 | | | | | | | | | |  |

**Supplementary Table 4.** Pearson correlation coefficients for sleep-related digital biomarkers and patient-reported outcomes. No adjustment for multiple comparisons was performed.

|  | **KCCQ: Summary score** | **KCCQ: Clinical Score** | **KCCQ: Physical limitations** | **KCCQ: Quality of life** | **KCCQ: Self-efficacy** | **KCCQ: Social limitations** | **KCCQ: Total Symptom** | **EQ 5D-5L Utility** | **EQ 5D-5L**  **VAS** |
| --- | --- | --- | --- | --- | --- | --- | --- | --- | --- |
| Longest sleep period | 0.135* | 0.130* | 0.137* | 0.110* | 0.087 | 0.144* | 0.107* | 0.075 | 0.041 |
| Naps duration | 0.070 | 0.052 | 0.066 | 0.046 | 0.065 | 0.069 | 0.042 | 0.102* | 0.031 |
| Sleep efficiency | 0.211* | 0.232* | 0.242* | 0.131* | 0.099* | 0.205* | 0.202* | 0.147* | 0.154* |
| Sleep  events | -0.192* | -0.155* | -0.162* | -0.174* | -0.024 | -0.214* | -0.136* | -0.109* | -0.140* |
| Sleep interval duration | -0.064 | -0.030 | -0.016 | -0.091 | 0.012 | -0.062 | -0.039 | -0.055 | -0.107* |
| Sleep onset latency | -0.104* | -0.114* | -0.123* | -0.098* | 0.024 | -0.102* | -0.096 | -0.112* | 0.037 |
| Time first WASO | 0.005 | -0.015 | -0.032 | 0.030 | 0.039 | 0.017 | -0.004 | 0.026 | 0.006 |
| Total sleep duration | 0.082 | 0.113* | 0.130* | 0.006 | 0.081 | 0.096 | 0.086 | 0.067 | 0.022 |
| * *P* < 0.05 | | | | | | | | | |
